# Fabrication of MoSe_2_ nanoribbons via an unusual morphological phase transition

**DOI:** 10.1038/ncomms15135

**Published:** 2017-05-04

**Authors:** Yuxuan Chen, Ping Cui, Xibiao Ren, Chendong Zhang, Chuanhong Jin, Zhenyu Zhang, Chih-Kang Shih

**Affiliations:** 1 Department of Physics, University of Texas at Austin, Austin, Texas 78712, USA; 2 International Center for Quantum Design of Functional Materials (ICQD), Hefei National Laboratory for Physical Sciences at the Microscale, and Synergetic Innovation Center of Quantum Information and Quantum Physics, University of Science and Technology of China, Hefei, Anhui 230026, China; 3 State Key Laboratory of Silicon Materials, School of Materials Science and Engineering, Zhejiang University, Hangzhou, Zhejiang 310027, China

## Abstract

Transition metal dichalcogenides (TMDs) are a family of van der Waals layered materials exhibiting unique electronic, optical, magnetic and transport properties. Their technological potentials hinge critically on the ability to achieve controlled fabrication of desirable nanostructures, such as nanoribbons and nanodots. To date, nanodots/nanoislands have been regularly observed, while controlled fabrication of TMD nanoribbons remains challenging. Here we report a bottom-up fabrication of MoSe_2_ nanoribbons using molecular beam epitaxy, via an unexpected temperature-induced morphological phase transition from the nanodot to nanoribbon regime. Such nanoribbons are of zigzag nature, characterized by distinct chemical and electronic properties along the edges. The phase space for nanoribbon growth is narrowly defined by proper Se:Mo ratios, as corroborated experimentally using different Se fluxes, and supported theoretically using first-principles calculations that establish the crucial role of the morphological reconstruction of the bare Mo-terminated edge. The growth mechanism revealed should be applicable to other TMD systems.

## Abstract (web summary)

The unique electronic properties of two-dimensional materials are determined not only by their shape, but also the precise atomic arrangement of atoms along edges. Here, Chen *et al*. have developed a bottom-up epitaxial growth of MoSe_2_ nanoribbons that controls both geometry and edge states.

As the first and arguably most important member of the two-dimensional (2D) materials family, pristine graphene possesses exotic intrinsic properties^1^,^2^,^3^,^4^ and properly tailored graphene nanostructures are further expected to exhibit various emergent properties of potential technological significance^5^,^6^,^7^,^8^. One compelling example is graphene nanoribbons, which may develop tunable bandgaps^9^,^10^, support robust edge states^11^ and display half-metallicity under an external electrical bias^12^. For these very reasons, various innovative methods have been developed to achieve controlled fabrication of graphene nanoribbons, including both bottom-up^5^,^13^,^14^,^15^,^16^ and top-down^6^,^10^,^17^,^18^,^19^ approaches, enabling the revelation of rich and intriguing physical phenomena^20^,^21^,^22^.

Beyond graphene, transition metal dichalcogenide (TMD) materials have emerged as another important class of 2D systems with inherently different physical properties, most notably the existence of intrinsic bandgaps^23^,^24^,^25^ and much stronger spin–orbit and spin–valley coupling effects^26^,^27^,^28^. When reduced to nanoribbon geometries, the very compound nature of the TMD systems further introduces more complexities when compared with their graphene counterparts. To date, only limited successes have been demonstrated in the fabrication of TMD nanoribbons using top-down approaches^29^,^30^; a viable bottom-up approach for controlled mass production of high-quality TMD nanoribbons remains to be discovered. This standing obstacle severely hinders potential advances in exploring their exotic electronic^31^, magnetic^32^,^33^, optical^34^,^35^ and catalytic^36^ properties for device applications.

Here we report the discovery of an unusual nanoribbon growth mode in molecular beam epitaxy (MBE) growth of MoSe_2_ on a graphite substrate. By properly controlling the substrate temperature and Se over-pressure, the MoSe_2_ atomic layers undergo a three-stage shape transformation: from fractal to compact 2D nanoislands, and eventually to nanoribbons, in contrast to the traditional two-stage growth behaviour involving only the transformation from the fractal to compact regime. Measurements using *in situ* scanning tunnelling microscopy/spectroscopy (STM/STS) and *ex situ* transmission electron microscopy (TEM) reveal that the nanoribbons extend in the zigzag direction, with distinctly different edges whose ideally bulk truncated forms are commonly called Mo- and Se-terminated^33^. Locally, the Se-terminated edge contains more kinks than the Mo-terminated edge. In addition, the electronic transition from the core of the nanoribbon to two different zigzag edges shows significant asymmetry. Synergistic efforts between theory and experiment further reveal that the Se:Mo flux ratio during MBE growth plays a central role in controlling the nanoribbon formation, favouring ribbon growth at lower Se flux, and the underlying reason can be attributed to different morphologies and energetics of the zigzag and armchair edges at different Se:Mo ratios. Such a morphological phase transition in MoSe_2_ nanoribbon growth is also expected to be qualitatively transferrable to other van der Waals (vdW) substrates as well as other TMD materials.

## Results

### Temperature-dependent growth morphology of MoSe_2_

**Figure 1:**
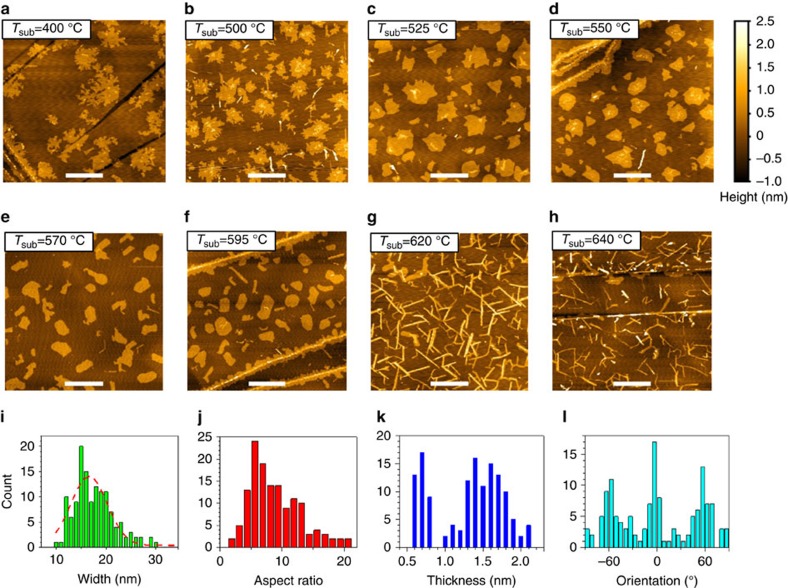
Temperature-dependent morphology and statistical analysis of MoSe_2_. (**a**–**h**) AFM images of MoSe_2_ grown at a series of *T*_sub_ with a three-stage shape transformation from fractal (**a**,**b**) to compact 2D nanoislands (**c**–**f**) and eventually to nanoribbons (**g**,**h**). Scale bars (white), 500 nm (**a**–**h**). **a**–**h** share the same colour scale for height, shown to the right of **d**. (**i**–**l**) Statistical analysis of the ribbon width, aspect ratio, thickness and orientation of the nanoribbons shown in **g**. The dashed red curve in **i** represents the fitted Gaussian distribution with an average value of 17 nm and a s.d. of 7 nm.

Monolayer (ML) MoSe_2_ has been successfully grown using MBE on various vdW substrates^25^,^37^,^38^,^39^,^40^. Our MBE growth of MoSe_2_ on highly oriented pyrolytic graphite (HOPG) could take place over a large range of substrate temperature (*T*_sub_), and the morphology of the MoSe_2_ flakes shows strong *T*_sub_ dependence. Figure 1a–h shows a sequence of ambient atomic force microscopy images of MoSe_2_ grown at different *T*_sub_ but at a fixed nominal Se:Mo flux ratio of 10:1. At *T*_sub_<500 °C, the MoSe_2_ flakes have fractal shapes, largely as a result of insufficient adatom diffusion around the island edges; at an intermediate *T*_sub_ (between 500 and 600 °C), MoSe_2_ forms 2D compact islands^41^,^42^,^43^. Many of the compact islands or nanodots are of triangular or hexagonal shapes, with well-defined corners of 60°, 90°, 120° and 150°. (See details in Supplementary Fig. 1, and the underlying growth mechanisms leading to these specific shapes will be discussed later.) When the growth temperature rises within this range, the compact islands become more and more isotropic, with increasing numbers of shorter straight edges and more smeared corners. Thus far, this temperature-dependent growth mode can be well understood within the contexts of shape transformations in nonequilibrium growth of surface-based nanostructures^41^,^42^,^43^.

When *T*_sub_ is even higher, a completely different morphology, nanoribbon, becomes dominant. As shown in Fig. 1g, when *T*_sub_=620 °C, nanoribbons with well-defined orientations become quite evident. As the temperature is raised further (for example, Fig. 1h for *T*_sub_=640 °C), the nanoribbon density becomes much lower and individual nanoribbons are not as straight as those at 620 °C. At even higher temperatures, one no longer can observe any growth of MoSe_2_ at all. The statistical analysis of the size/shape and orientation of the nanoribbons grown at *T*_sub_=620 °C is presented in Fig. 1i–l. The width of the nanoribbons matches with a Gaussian distribution, possessing an average value of 17 nm and a s.d. of 7 nm as shown in Fig. 1i. The aspect ratio is most probably between 4 and 14, with exceptions as high as 20. The thickness distribution indicates that the ribbons are mostly ML and bilayer (BL), and the ML ribbons have better defined thickness than the BL ribbons. The well-defined orientations of the nanoribbons are 60° apart, clearly manifesting the threefold symmetry of the substrate. More statistical analyses on only the ML nanoribbons are given in Supplementary Fig. 2. Such a morphological phase transition from the compact to elongated shape is unusual and unexpected.

### Lattice and electronic structures of the MoSe_2_ nanoribbons

**Figure 2:**
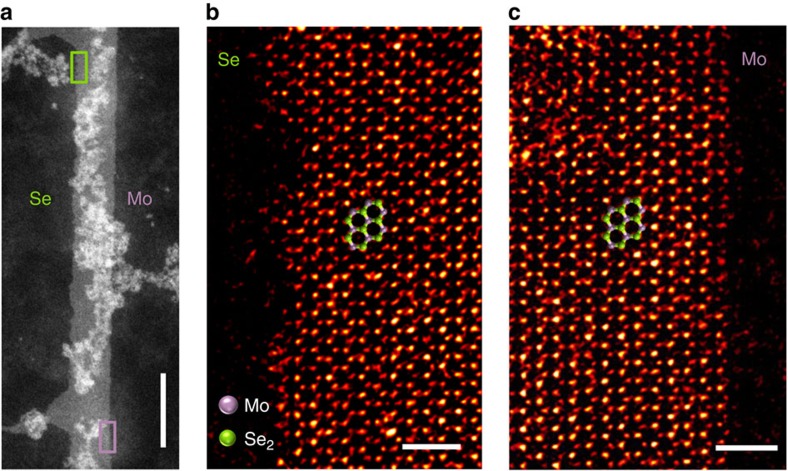
Atomic properties of MoSe_2_ nanoribbons. (**a**) Large-scale TEM image of a nanoribbon. Scale bar (lower right corner), 20 nm. Atomic-resolution images in the highlighted green and purple rectangles are shown in **b** and **c**, respectively. The edges are characterized as zigzag edges, with the left edge (in **b**) being Se-terminated, while the right (in **c**) being Mo-terminated. In both **b** and **c**, four-ring fragments of the ball-and-stick honeycomb lattice of MoSe_2_ (purple, Mo; green, Se) are superposed onto the TEM images as visual guides. Scale bars, 1 nm (**b**,**c**).

**Figure 3:**
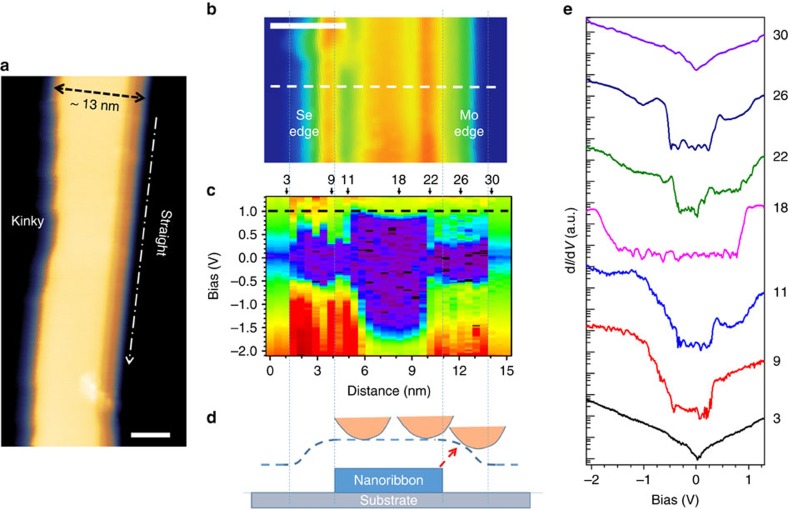
Electronic properties of MoSe_2_ nanoribbons. (**a**) STM topography image of a nanoribbon, taken at the sample bias of *V*_b_=+3 V and tunnelling current of *I*_tunnel_=10 pA. Scale bar (lower right corner), 5 nm. (**b**) STM topography of a nanoribbon segment, taken at *V*_b_=+1.0 V and *I*_tunnel_=5 pA. The left edge has more kinks and is determined to be Se-terminated, while the straighter right edge is Mo-terminated. Scale bar (upper left corner), 5 nm. (**c**) Colour-scaled d*I*/d*V* mapping across the nanoribbon taken along the white dashed line in **b** with a separation of 0.5 nm between adjacent sampling points. The range of bias sweep is from −2.1 to 1.3 V. The black dashed line is located at +1.0 V, the bias applied in **b**. The asymmetry in the electronic structures of the two edges is evident. From left to right, there are three electronically distinct areas: Se-terminated edge, core and Mo-terminated edge. (**d**) A schematic illustration of the edge broadening mechanism. (**e**) Representative individual d*I*/d*V* spectra at some typical areas across the MoSe_2_ nanoribbon and on the HOPG substrate, as marked by the arrows in **c**. The spectra lines are plotted in logarithmic scale, with proper offsets.

To identify the morphological nature of the MoSe_2_ nanoribbons, their atomic and electronic structures have been characterized by TEM and STM/STS. Typical zoom-in views of the nanoribbons are shown in Figs 2a and 3a. We notice that one edge of a typical nanoribbon is usually straighter, while the other one contains more kinks. This asymmetric feature of the two different edges is also displayed in two more STM images shown in Supplementary Fig. 3. As mentioned earlier, ideally bulk truncated TMD nanoribbons within the stable 1H structure have two different types of zigzag edges, one Mo-terminated and the other Se-terminated, but only one type of armchair edge^32^,^33^. Therefore, the asymmetry of the edge morphology strongly suggests that the long and distinctly different edges of the nanoribbons are zigzag edges. Indeed, the atomic-resolution images in Fig. 2b,c evidently show the honeycomb pattern, which confirms the lattice structure of the MoSe_2_ nanoribbons to be still the stable 1H phase instead of the metastable 1T or 1T′ phase^28^. This observation implies that there is no structural phase transition accompanying the morphological phase transition, the latter resulting in the formation of the nanoribbons. Moreover, from the atomic registry determined by the brightness of the atoms, it is inferred that the long edges of the ribbons are all zigzag edges, with the straighter edges to be Mo-terminated, while the more kinky edges to be Se-terminated edges. A closer look also reveals that along the Se-terminated edges, there exit micro-facets, bordered by additional (or energetically preferred) Mo-terminated edges, as shown in Fig. 2b. It is also noticed that there are non-crystalline clusters on both the nanoribbons and the substrates. The BL thickness shown in Fig. 1k is likely complicated by such clusters, which cannot be distinguished by the ambient AFM. Energy-dispersive X-ray spectroscopy has been applied to identify the chemical composition of the clusters, shown in Supplementary Fig. 4. Evidently, the Se signal only appears in the crystalline nanoribbon area, while the Mo signal appears in both the nanoribbon and cluster. These observations suggest that, during the nanoribbon growth, there were extra Mo atoms on the HOPG surface, despite the nominal Se:Mo ratio that was deliberately kept at 10:1.

STM/STS is utilized to characterize the electronic structure of the MoSe_2_ nanoribbons, and the results are shown in Fig. 3. For a ribbon of average width as in Fig. 3b, the differential tunnelling conductance (d*I*/d*V*) spectra^44^,^45^,^46^ on 32 successive points across the ribbon were taken and mapped out in colour code in Fig. 3c to visualize the substantial variations in the local electronic structure. The purple colour represents zero or negligible d*I*/d*V*, while the warmer the colour, the higher the d*I*/d*V* value. Obviously the mapping contains a non-uniform gapped region in the middle (from spectrum #4 to #29) corresponding to the nanoribbon, and two gapless regions on both sides (from spectrum #1 to #3 and from #30 to #32) corresponding to the HOPG substrate. A few representative spectra out of the measure data set are stacked and shown in Fig. 3e with proper offsets. From left to right on the gapped region, there are three electronically distinct areas: Se-terminated edge, core and Mo-terminated edge. Here we note that determination of the bandgap of a given TMD system demands extreme care due to the fact that the valence band maximum at the K point with a very large parallel momentum has a much smaller tunnelling probability than the state at the Γ point^38^. In the case of MoSe_2_, the valence band maximum at the K point occurs roughly at 0.4 eV above the Γ point. If the d*I*/d*V* data encompass a broad enough dynamic range (more than three orders of magnitude), then the threshold near the K point can hardly be identified in typical linear-scale plots, but can be readily pinpointed when the d*I*/d*V* data are plotted in logarithmic scale^37^,^45^,^46^. Using the spectrum #18 in Fig. 3e, the bandgap of the core region can then be determined as ∼2.28 eV, the same as the bandgap of ML 2D flakes within the 1H phase^37^,^38^ (see details in Supplementary Fig. 5). This observation once again confirms that there is no structural phase transition in the nanoribbons.

Before going into detailed discussions of the electronic structures at the edges, we need to first identify the physical location of the edges. Because the nanoribbon is physically 0.7 nm higher than the graphite substrate, the apparent width in the STM image is likely widened due to the finite radius of the probe, as schematically illustrated in Fig. 3d. Namely, as the probe scans across the edge, there is a transitional width where the tunnelling still occurs between the edge and the sidewall of the tip, as indicated by the red dashed arrow. It is quite common to have a transitional width of 2–3 nm, giving rise to an apparent widening of the edge in both the STM image and STS mapping. This explains why in Fig. 3c, the electronic structures of the edges expand over 3 nm (spectra #4–#11 and #22–#31), much wider than the calculated ones^33^. We have used different tips and found that the width of this extended edge region varies, confirming that this extended edge width is due to the probe shape.

Spectroscopically, the fluctuations observed in the regions of spectra #4–9 and #24–29 likely reflect the fact that the tunnelling occurred between the edge and different points at the tip sidewall. The actual edge region is likely only ∼1 nm. Therefore, we use the spectra #11 and #22, taken right after the transition from the bulk region, to represent the bandgaps of the edges. Following the same approach as described above, we find that the Se-terminated edge has a gap of 0.64 eV, while the Mo-terminated edge has a gap of 0.36 eV (see Supplementary Fig. 6 for more details).

At this point it is worthwhile to note that, almost all experimental results, including the STS data in the present study, show a finite bandgap at the edge^46^; in contrast, most theoretical calculations would predict a metallic^32^,^33^ edge. Such an inconsistency might be due to the lacking of accurate atomic models of the zigzag edges. It is noted that with particular edge reconstructions and passivation, a significant bandgap opening has been proposed in a recent density functional theory (DFT) work^47^. Depending on the detailed models, the gap can be predicted to be as large as 0.7–1.2 eV. Indeed, the possibility of having additional Se passivation after growth is highly likely, because we annealed the sample in the Se flux after the growth for ∼15 min. This post-growth annealing may change the final termination. On the other hand, it does not change the fact that during the growth of the nanoribbons, the system is under the Se-poor condition. Moreover, in our system, non-negligible charge transfer may occur between the semimetal substrate and the edges, which might also lead to a zero conductance at the Fermi level.

It is worthwhile to emphasize that, overall, both the chemical purity and morphological quality of the nanoribbons fabricated here using the bottom-up self-assembly approach are clearly superior to those achieved using the more intrusive top-down approaches of TEM-based cutting of pristine TMD MLs.

### Conjecture on the morphological transformation mechanism

In attempting to reveal the likely underlying mechanism for the morphological phase transition from the compact islands to the nanoribbons, we note that, ultimately, the evolution of the island shape is dictated by the relative energetics and growth rates of the different and competing edge structures, namely, bare zigzag (Mo-terminated and Se-terminated) and armchair edges and their passivated counterparts. If the bonding configurations of these edges remained the same throughout the vastly different growth conditions, a higher temperature would only lead to islands with more isotropic shapes^43^. The growth behaviour in the sequence from Fig. 1c to f indeed follows this expectation. Then, what causes the marked transformation from the compact 2D islands to the nanoribbons at 620 °C? The most probable reason is that the relative energetics and growth rates between the different edges have been altered at the transition temperature.

Here we recall that MBE growth of MoSe_2_ needs to be carried out under a high Se:Mo flux ratio to keep Se atoms available on the surface for growth (with super-saturated Se due to its high vapour pressure). At a constant Se flux, a higher substrate temperature would actually imply a lower concentration of available Se adatoms at the growth front of the 2D islands, because the binding energy of Se is much lower than that of Mo. In fact, the energy-dispersive X-ray spectroscopy results in Supplementary Fig. 4 imply that the actual amount of Se was even insufficient to react with all the Mo atoms on the HOPG surface. We therefore conjecture that the available Se concentration on the surface must have played a crucial role in modifying the bonding configurations of different edge structures and thus altering their relative energetics and growth rates. In the following, this conjecture is further investigated both experimentally and theoretically.

### Experimental validation of the conjecture

**Figure 4:**
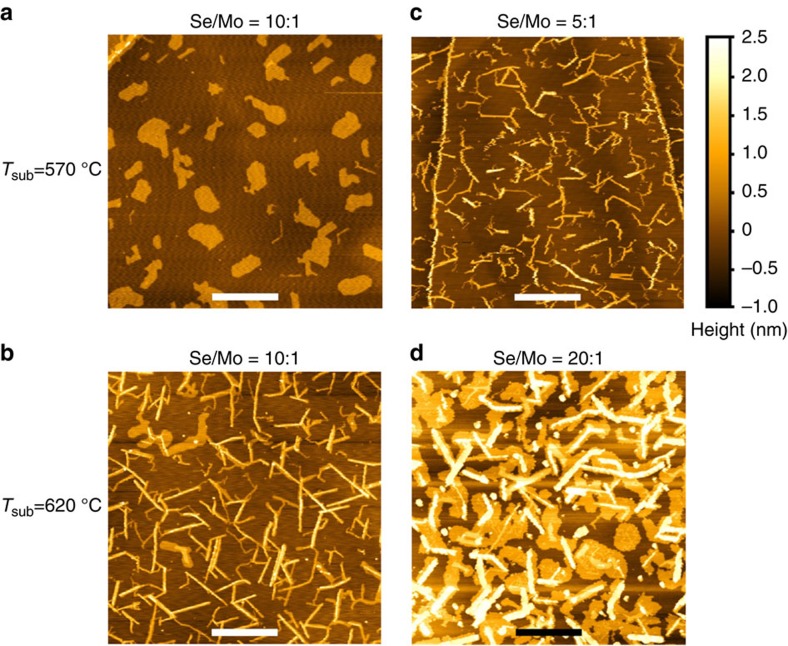
Se:Mo ratio-controlled morphological phase transition. (**a**,**b**) Same AFM images of MoSe_2_ at the Se:Mo flux ratio of 10:1 as Fig. 1e,g, respectively. (**c**) AFM image of MoSe_2_ grown at the same *T*_sub_ as in **a**, but with the Se:Mo flux ratio reduced by one half to 5:1. (**d**) AFM image of MoSe_2_ grown at the same *T*_sub_ as in **b**, but with the Se:Mo flux ratio doubled to 20:1. Scale bars (white for **a**–**c** and black for **d**), 500 nm. **a**–**d** share the same colour scale for height, shown to the right of **c**.

We now independently control the Se:Mo flux ratio, and compare the new growth mode with the earlier ones. It is evident that the transition temperature from the 2D (or compact) to 1D (or elongated) growth mode can indeed be altered by the nominal Se:Mo flux ratio. At 570 °C, which fell into the 2D compact growth regime earlier (Fig. 4a), nanoribbons can now also form when the Se pressure is lowered by one half, to Se:Mo=5:1 (Fig. 4c). Furthermore, at 620 °C, which fell into the nanoribbon growth regime earlier (Fig. 4b), 2D compact islands now become the dominant morphology when the Se pressure is doubled, to Se:Mo=20:1 (Fig. 4d). These definitive observations corroboratively verify the important role of Se adatoms in triggering the marked morphological transformation, favouring nanoribbon growth at lower Se concentrations.

### Atomistic growth mechanisms of the MoSe_2_ nanoribbons

To gain further insights into the atomistic growth mechanisms of the nanoribbons, we use first-principles calculations within DFT (see Methods for details) to compare the energetics of the zigzag and armchair nanoribbons under the two limiting cases of Se-poor and Se-rich conditions. In either case, we model the zigzag or armchair nanoribbons with supercells that contain the same numbers of Mo and Se atoms, allowing direct comparison of their relative stability by their total energy difference. To reveal the growth mechanism based on the DFT results, we would like to emphasize the following three points related to the growth processes. First, the evolution of the MoSe_2_ island shape is dictated by the relative energetics and growth rates of the different and competing edge structures that include bare zigzag (Mo-terminated and Se-terminated) and armchair edges and their passivated counterparts. Second, an edge with lower energy is more stable, thereby grows slower in the normal direction and faster along the edge direction than that with higher energy. Third, the growth temperature and Se:Mo flux are two main control knobs, which have somewhat equivalent effects on the growth.

**Figure 5:**
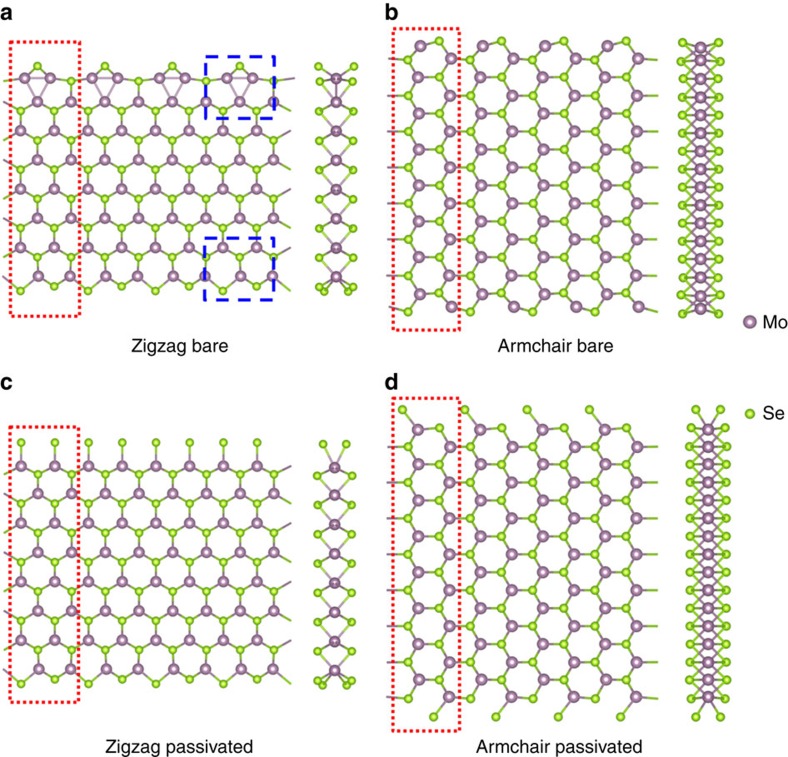
Optimized atomic structures and energy differences of different MoSe_2_ nanoribbons. (**a**,**c**) Structures of zigzag nanoribbons without and with extra Se dimers along the edges, respectively. (**b**,**d**) The corresponding structures of armchair nanoribbons. Both top and side views are shown for the nanoribbons. The atoms included in the supercell calculations in each case are indicated by the dotted rectangle. The (2 × 1) reconstruction of the bare Mo- or Se-terminated edge is shown by the respective dashed rectangle in **a**. The energy difference in the upper case (without extra Se dimer passivation) is 0.85 eV per supercell, while that in the lower case is 0.10 eV per supercell.

As illustrated in Fig. 5a,b under the Se-poor condition defined by unpassivated Mo-terminated edges, a global (2 × 1) reconstruction along both the Mo- and Se-terminated edges occurs for the zigzag nanoribbons, while the edge Mo and Se atoms tend to shift only slightly for the armchair nanoribbons^33^. For the zigzag nanoribbon, the (2 × 1) reconstruction along the Mo-terminated edge is via place exchange, with substantial inward displacements of all the first-row Mo atoms and corresponding outward displacements of half of the second-row Se atoms, making the edge Mo atoms all effectively passivated by the displaced Se atoms rather than by extra Se adatoms^33^. The (2 × 1) reconstruction of the Se-terminated edge is significantly milder than that at the Mo-terminated edge, characterized by slight local place readjustments of the edge atoms. Such edge reconstructions substantially reduce the total energy of the zigzag nanoribbon by 0.85 eV per supercell compared to the armchair nanoribbon. Therefore, a rectangular island of MoSe_2_, containing the opposite zigzag edges and armchair edges simultaneously, prefers longer zigzag edges rather than the armchair ones, that is, the higher-energy armchair edges grow much faster than the zigzag edges, resulting in the ribbon structures as observed experimentally. For islands of different initial shapes, this nanoribbon growth mechanism can also be operative, as shown in Supplementary Fig. 7.

Within this growth mechanism, the two edges of the MoSe_2_ nanoribbons should be the two different zigzag edges, namely, Mo-terminated edge and Se-terminated edge, which have been confirmed by the TEM images of the nanoribbon shown in Fig. 2b,c. Our DFT calculations also show that the energy gain along the reconstructed Mo-terminated edge is 0.82 eV per formula unit relative to the unreconstructed case, while the energy gain for the reconstructed Se-terminated edge is 0.16 eV per formula unit. The distinct energy difference should provide an important basis for differentiating such edges in the experiments. In particular, the more stably reconstructed Mo-terminated edge becomes straighter, while the less stable Se-terminated edge contains more kinks, as displayed in Fig. 3a.

In contrast, under the Se-rich condition, the excess Se adatoms can effectively passivate the edge Mo atoms. Figure 5c,d shows the structures of the zigzag and armchair nanoribbons with an extra Se dimer added at each edge Mo atom, while the inner atoms essentially keep their respective bulk-terminated positions. The total energies of the fully passivated zigzag and armchair nanoribbons are found to be much closer, with the former lower by only 0.10 eV per supercell. In this case, the growth rates of the zigzag and armchair edges are also expected to be nearly the same, thereby favouring compact structures of the MoSe_2_ islands, as shown in Fig. 1c–f. Here it is also worthwhile to point out that the corners of 60°, 90°, 120° and 150° in the compact structures of the MoSe_2_ islands in Fig. 1c–f and highlighted in Supplementary Fig. 1 for the case at or near the transition, originate from the inter-junctioning of two identical types of zigzag or armchair edges, one zigzag and one armchair edge, two different types of zigzag edges or two identical types of armchair edges, and one zigzag and one armchair edge, respectively. The precise shapes of the compact MoSe_2_ islands depend on the delicate competitions between the slightly different growth rates of the armchair edge and two zigzag edges.

Before closing, we also briefly discuss the validity of ignoring the HOPG substrate when developing the dominant nanoribbon growth mechanism based on the DFT results. The substrate effect should definitely play a role in determining the orientations of the MoSe_2_ nanoribbons or islands during the growth as shown in our experiments, but may not influence in an essential way on the morphological phase transition from the compact islands to the nanoribbons. The reasons why we did not specifically take the substrate effects into account when proposing the growth mechanism for such a morphological phase transition are as follows. First, the interaction between the HOPG substrate and MoSe_2_ is the relatively weaker vdW interaction, rather than the much stronger chemical bonding in more traditional growth systems. In the initial stage, the nucleation of MoSe_2_ assisted by HOPG will choose some preferred directions due to the threefold symmetry of the substrate. In the growing stage, the sustainable Mo and Se atoms reach at and attach to the growth front of the MoSe_2_ islands, which are thereby enlarged. Due to the vdW interaction between the edge (or growth front) of the MoSe_2_ and HOPG, the substrate will not influence much on the specific morphology of the MoSe_2_ systems, for example, whether they grow into compact islands or nanoribbons. Second, based on our detailed DFT calculations, the total energy of the bare zigzag-edged nanoribbon is much lower than that of the armchair-edged one by 0.85 eV per supercell. It is noted that the energies of the inner atoms for the zigzag and armchair nanoribbons should be quite similar; therefore, the energy difference is mainly contributed from the edge atoms. In particular, the edges within the supercells considered in our study (2 × 1 for the zigzag ribbons and 1 × 1 for the armchair ribbons, see Fig. 5) are very short along the ribbon direction, invoking a marked energy difference of 0.85 eV. Such a difference is unlikely to be substantially weakened by the vdW force of the substrate. Therefore, the growth mechanism proposed here should stay valid even if the substrate is considered, and the ribbon growth phenomena should also be expected for other related TMD systems on substrates that promote vdW growth.

In conclusion, tunable mass production of well-defined zigzag MoSe_2_ nanoribbons has been achieved for the first time experimentally, and the underlying mechanism for nanoribbon growth has been revealed through synergistic efforts between controlled experiments and first-principles calculations. Such a bottom-up fabrication scheme of nanoribbons should also be transferrable to other vdW substrates, and for other TMD materials. The ready access to TMD nanoribbons as achieved in this study is expected to enable substantial future explorations of the exotic electronic, magnetic, catalytic and transport properties of various TMD nanoribbons for potential technological applications.

## Methods

### Sample preparation via MBE

The HOPG substrate was exfoliated in ambient condition and transferred into the MBE system through load-lock. The high-purity Mo (99.95%) and Se (99.999%) elemental sources were evaporated from an electron-beam evaporator and an effusion cell, respectively, in an ultra-high vacuum chamber with a base pressure of 7.0 × 10^−11^ torr. The substrate was heated by filament radiation. All the MBE depositions were followed by 15-min annealing at the growth temperature with the Se flux maintained.

### TEM characterization and image simulations

The sample was transferred onto a TEM grid following a previous report with minor modifications^48^. The MoSe_2_ ribbons together with a few layers of graphite (∼1–2 nm in thickness) were first exfoliated from the substrate. Then, the samples were transferred onto the TEM grid after removing the glue used in the cleavage. Due to the finite thickness and relatively light mass of the carbon atoms, the electron scattering from the residual graphite layers is much weaker than those from the MoSe_2_ layer, making the substrate invisible. The TEM images were recorded with a probe-corrected Titan ChemiSTEM (FEI, USA), which was operated at an acceleration voltage of 200 kV. The probe current was set at 47 pA with a convergent angle of 22 mrad for illumination. The inner collection angle was adjusted to be 44 mrad to enhance the contrast of Se atoms. The experimental TEM images shown in the main text were processed with improved Wiener-Filtering to increase the signal-to-noise ratio for better display.

### STM and STS measurements

All STM/STS investigations reported here were acquired at 77 K in ultra-high vacuum with a base pressure <6.0 × 10^−11^ torr. Electrochemically etched W-tips were cleaned *in situ* with electron-beam bombardment. The tunnelling bias was applied to the sample. The d*I*/d*V* spectroscopy at a constant tip–sample distance was taken by using a lock-in amplifier with a modulation voltage of 10 mV and at a frequency of 914 Hz.

### DFT calculations

The DFT calculations were performed using the projector-augmented wave method^49^ implemented in the Vienna *ab initio* simulation package^50^,^51^. For the exchange-correlation functional, we used the generalized gradient approximation of Perdew, Burke and Ernzerhof^52^. A plane-wave basis set was adopted with an energy cutoff of 350 eV. In each supercell, the vacuum layers between two neighbouring ribbons are thicker than 16 Å along both the transverse and vertical directions of the ribbons. The one-dimensional Brillouin zone was sampled using a 32 × 1 × 1 Monkhorst–Pack K point mesh for all the ribbons considered. All the atoms were fully relaxed by the conjugate gradient algorithm until the residual forces were <0.01 eV Å^−1^. The HOPG substrate is not specifically considered in these calculations, an approach well justified, because its role is mainly on determining the overall orientations of the MoSe_2_ islands, rather than influencing on the morphological phase transitions.

### Data availability

The data that supports the findings of this study are available from the corresponding authors on request.

## Additional information

**How to cite this article:** Chen, Y. *et al*. Fabrication of MoSe_2_ nanoribbons via an unusual morphological phase transition. *Nat. Commun.* **8**, 15135 doi: 10.1038/ncomms15135 (2017).

**Publisher's note:** Springer Nature remains neutral with regard to jurisdictional claims in published maps and institutional affiliations.

## Supplementary Material

Supplementary Information Supplementary Figures.
