# Supplementary material for: Fabrication of MoSe2 nanoribbons via an unusual morphological phase transition
Source: Nat Commun. 2017 May 4;8:15135. doi: 10.1038/ncomms15135 (PMC5418606; doi:10.1038/ncomms15135)
Supplement: Supplementary Information — Supplementary Figures. [file ncomms15135-s1.pdf]

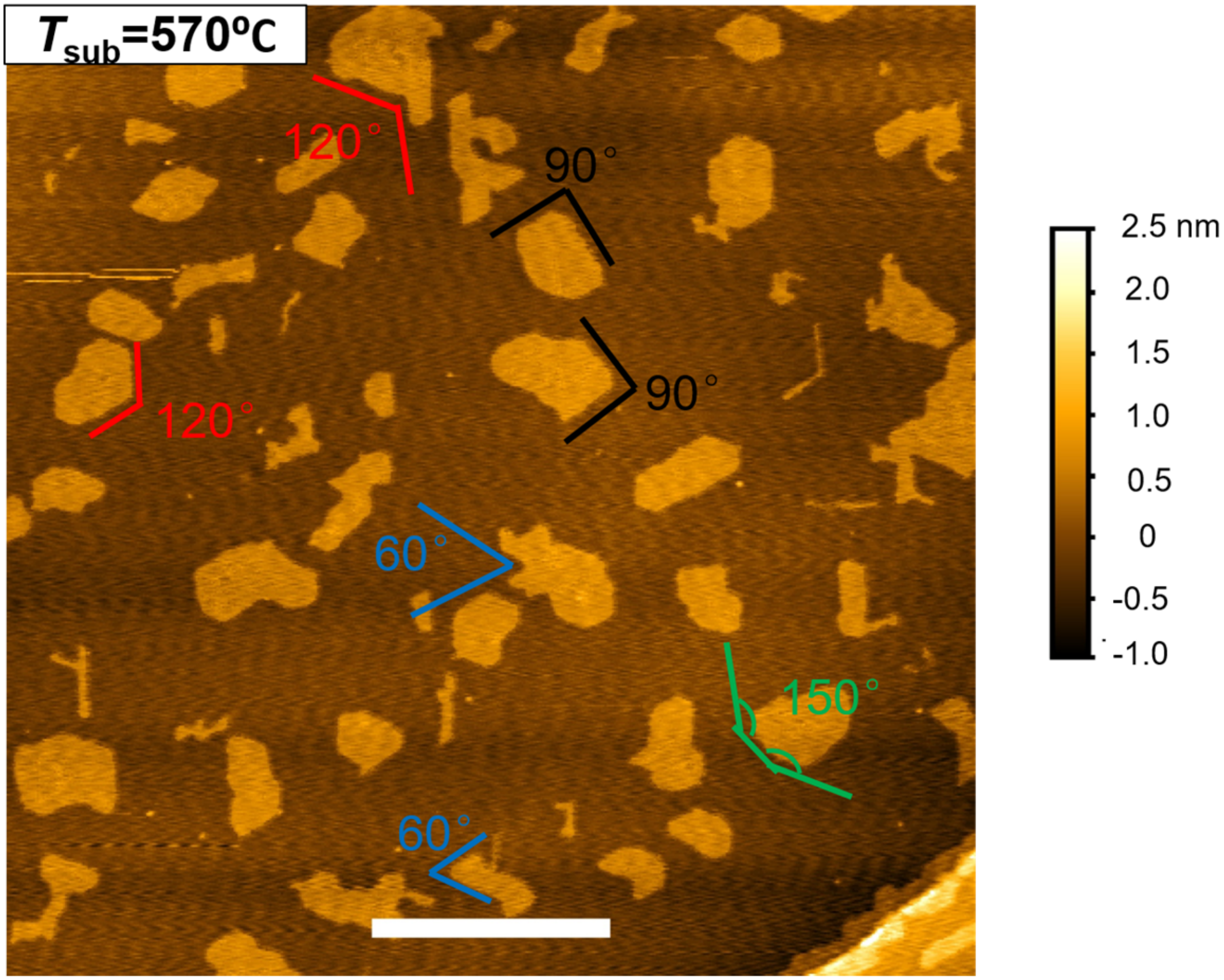

Supplementary Figure 1 | Typical angles of the corners of the 2D compact $MoSe_2$ islands. The scale bar at the bottom represents 500 nm. 60˚, 90˚, 120˚ and 150˚, originated from the inter-junctioning of different zigzag and/or armchair edges, are found to be the favorable angles. The precise shapes of the compact $MoSe_2$ islands depend on the delicate competitions between the slightly different growth rates of the armchair edge and two zigzag edges.

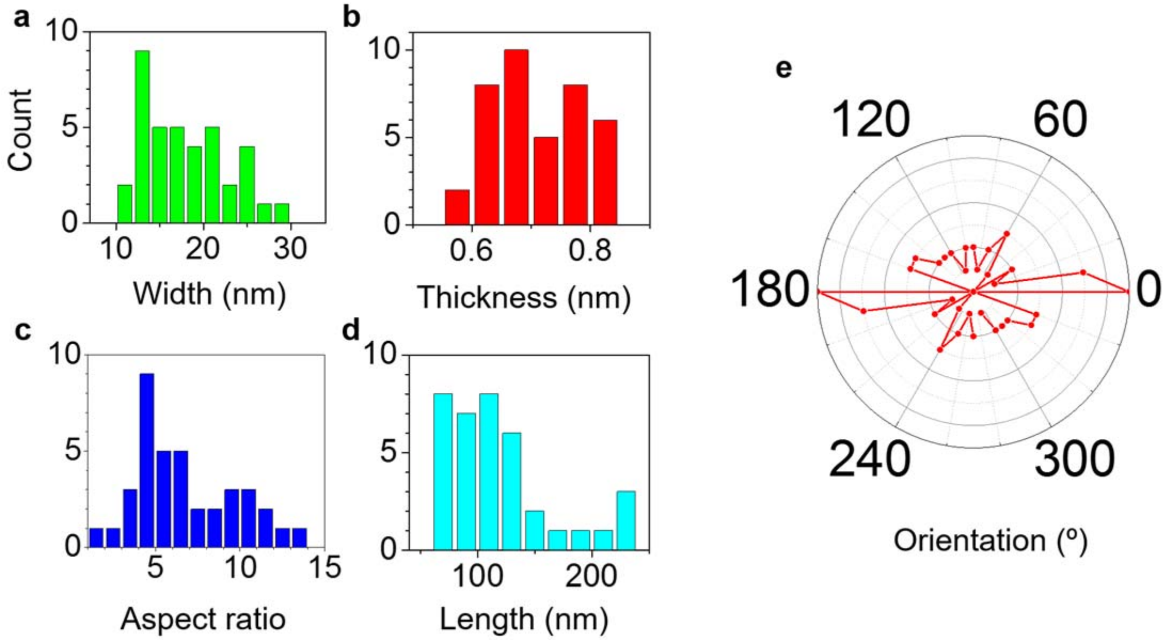

Supplementary Figure 2 | Statistical analysis of monolayer $MoSe_2$ nanoribbons. (a-d) Width, thickness, aspect ratio (length/width)**,** and length of the monolayer (ML) nanoribbons. (**e**) Orientation distribution of the ML nanoribbons in polar coordinates. The radian axis represents statistical counts with each solid grid illustrating 2 counts. For example, the count number for the direction of 0˚ is 7 counts. The direction of 0˚ was actually the direction of the parallel momenta of the impinging Mo atoms during the MBE growth. (Our heating stage does not rotate during the growth, so the relative parallel momentum of the Mo atoms stayed the same.) Such distribution is clearly anisotropic, and the favored directions are 30˚, 60˚ or 90˚ apart. Such anisotropy is expected to be introduced by the HOPG substrate.

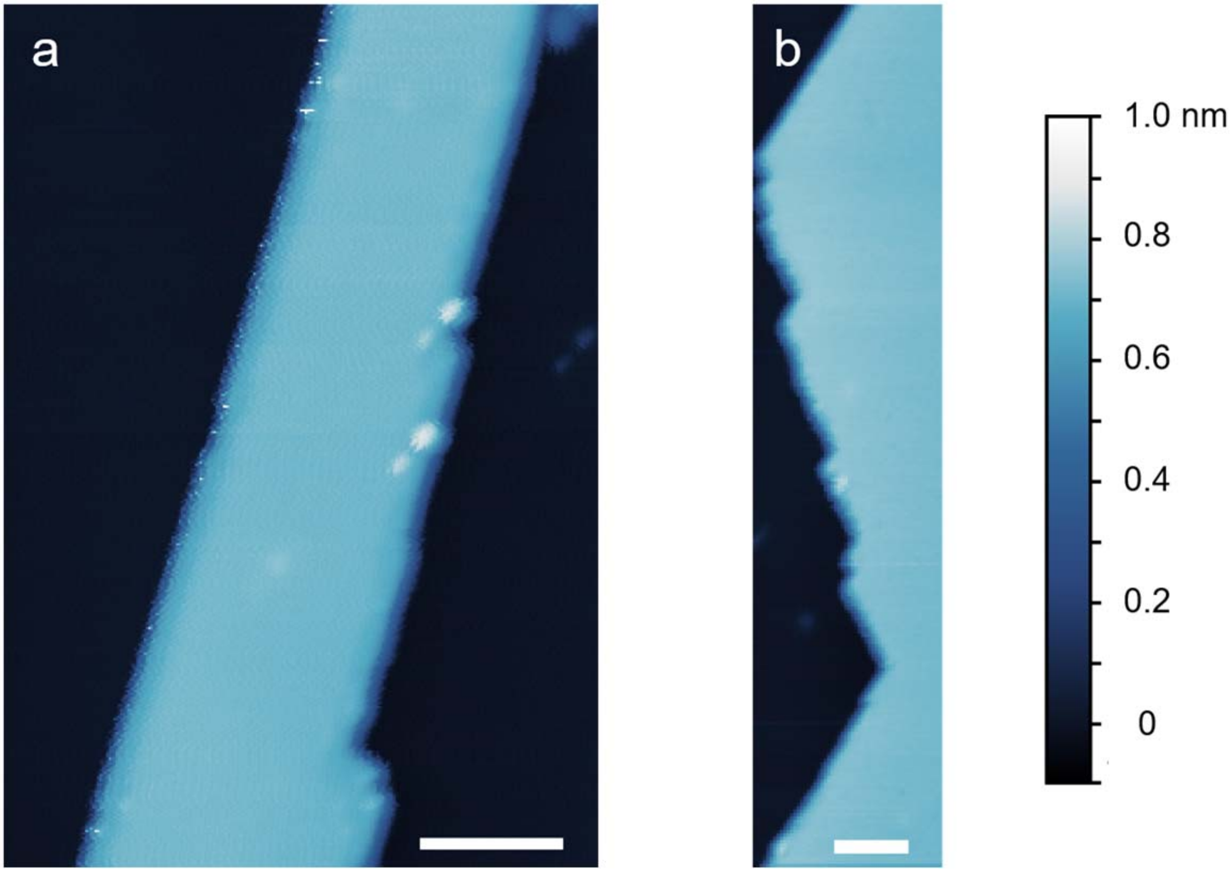

Supplementary Figure 3 | Evidence of asymmetric zigzag edges. (**a**) STM topography image of a monolayer $MoSe_2$ nanoribbon of about 15nm wide, with the left edge straight and the right edge kinky. The scale bar represents 10 nm. (**b**) STM topography image of the edges of a monolayer $MoSe_2$ 2D island found on the same batch of samples. The neighbouring edges with 120˚ angular separation are differently terminated zigzag edges. Such morphological difference was not observed on 2D islands grown at lower temperatures. The scale bar represents 5 nm. All images were taken at the sample bias of V_b = +2.5 V and tunnelling current of I_{tunnel} = 5 pA.

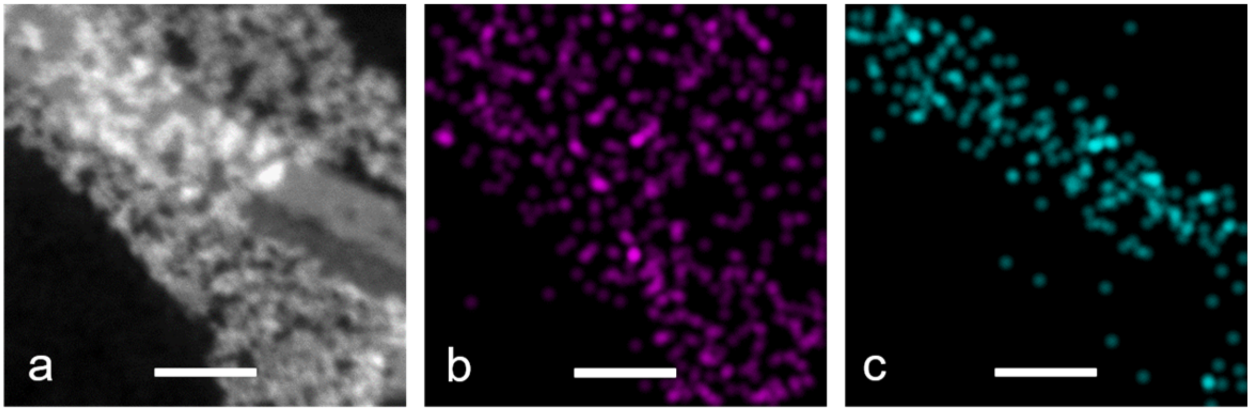

Supplementary Figure 4 | Morphology and chemical identifications of nanoribbon and non-crystalline clusters. (**a**) TEM image of $MoSe_2$ nanoribbon and non-crystalline clusters. (**b**,**c**) EDX maps of Mo and Se, respectively. Apparently, Mo existed in both the nanoribbon and the cluster region, while Se only existed in the nanoribbon. Therefore, the non-crystalline clusters seen in Fig. 2a were Mo clusters. The scale bars in all figures represent 5 nm.

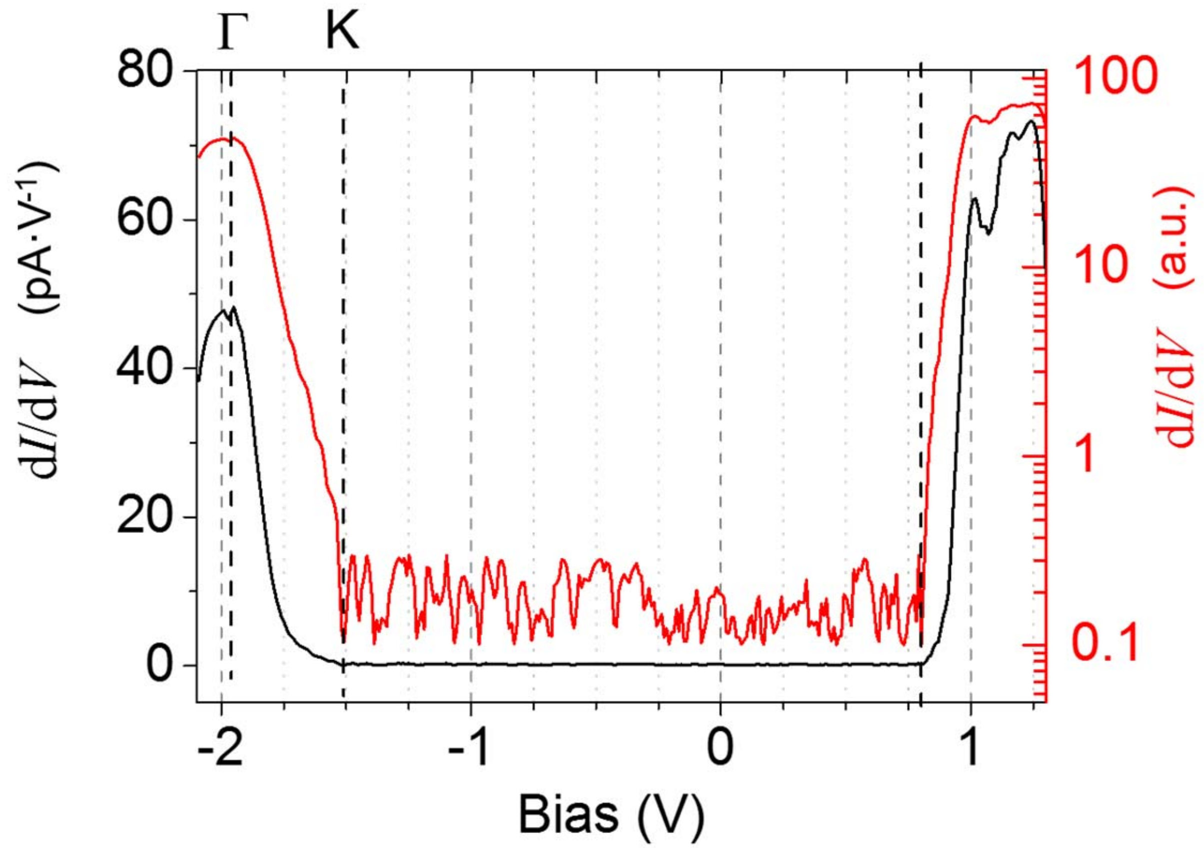

Supplementary Figure 5 | d*I*/d*V* spectra displayed in both linear scale and logarithmic scale. Displaying the spectrum in logarithmic scale increases the accuracy in determining the conduction band and valence band edges. It is necessary to offset the d*I*/d*V* curve by 0.1 in the logarithmic scale, to properly displace the spectrum, but such operation does not influence the accuracy in determining the band edges. The conduction band minimum (CBM) is 0.78 V, the valence band maximum (VBM) is -1.5 V, therefore the bandgap in the core of the ribbon is 2.28 eV.

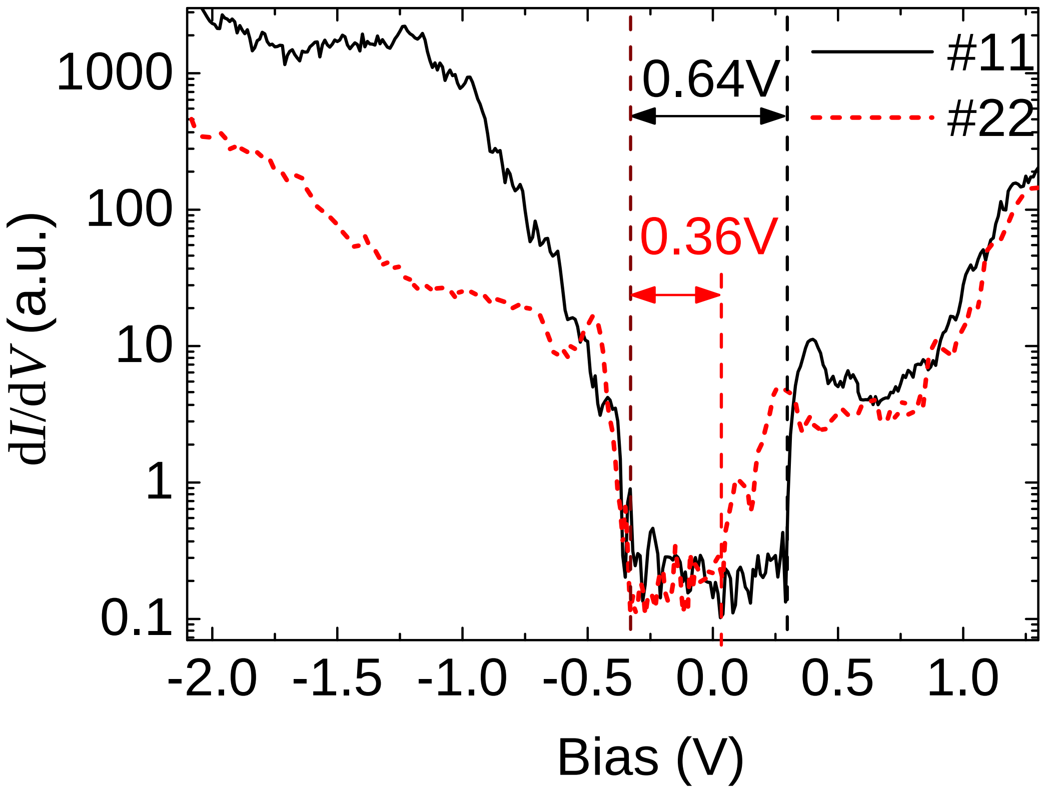

Supplementary Figure 6 | Asymmetric band structures of the zigzag edges. The Se-terminated edge (black, #11) has a gap of 0.64 eV and the Mo-terminated edge (red, #22) has a gap of 0.36 eV. Both bandgaps of the two edges are smaller than that of the core region.

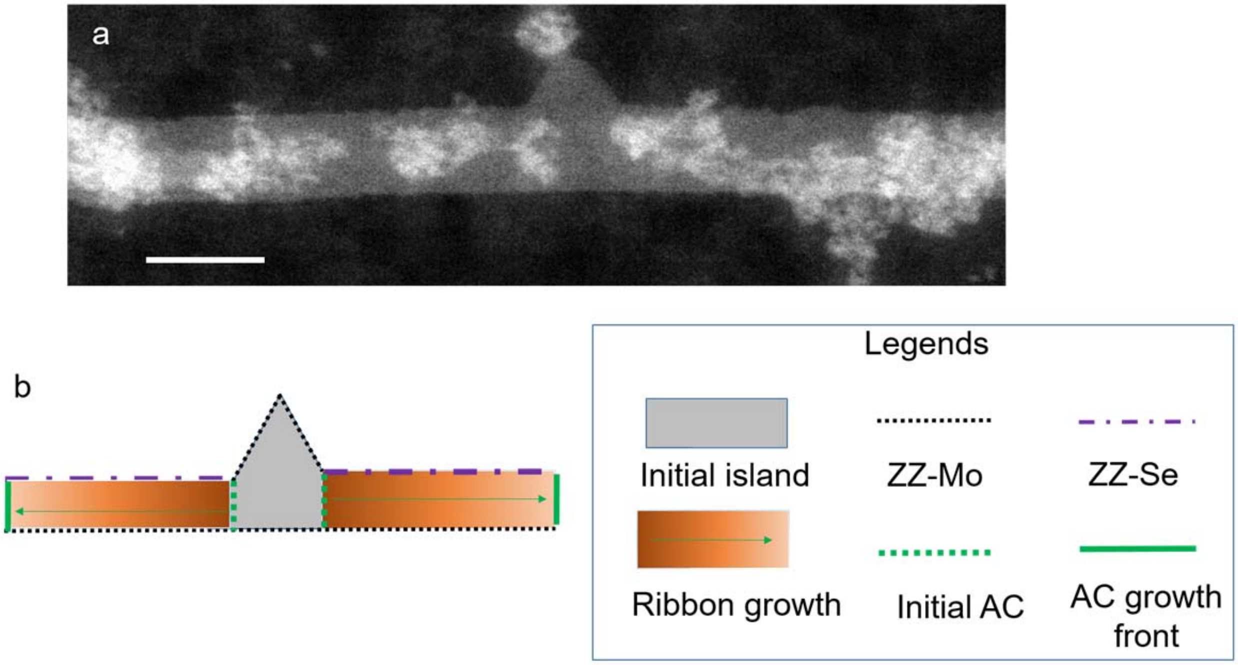

Supplementary Figure 7 | Illustration of nanoribbon growth initiated from a triangular cluster. (**a**) A nanoribbon with a triangular extrusion in the middle. The scale bar represents 20nm. (**b**) Schematic of this nanoribbon's growth. The initial pentagon can be seen as a triangular island with two corners cut off. The two edges perpendicular to the ribbon orientation (green dashed lines) are armchair (AC) edges, where the nanoribbon growth initiated. As the growth proceeds, AC edges do not grow longer, but work as the growth fronts, leaving two zigzag (ZZ) edges behind. In Se-deficient condition, such configuration maximizes the lengths of the reconstructed ZZ edges, so it is more stable than 2D compact islands.
